# Circulating exosomes carrying an immunosuppressive cargo may interfere with adoptive cell therapies in leukemia

**DOI:** 10.1186/2051-1426-3-S2-P60

**Published:** 2015-11-04

**Authors:** Michael Boyiadzis, Chang-Sook Hong, Theresa L Whiteside

**Affiliations:** 1 University of Pittsburgh, Pittsburgh, PA, USA

## Background

Adoptive immunotherapy, including transfer of activated NK cells, is currently under active investigation for patients with refractory and relapsed acute myeloid leukemia (AML). However, a highly immunosuppressive microenvironment sustained in part by tumor-derived exosomes, creates a major hurdle for adoptive immunotherapy. We recently reported that exosomes, virus-size (30-100nm) membrane-bound vesicles, represent one immunosuppressive mechanism operating in AML. We found high levels of exosomes in plasma of newly-diagnosed AML patients prior to any therapy. These exosomes carried an immunosuppressive cargo, including membrane-associated TGF-β1, PD-1, MICA/MICB and markers of myeloid blasts. Infusion of activated NK cells in patients with AML did not result in the expected recovery of NK cell activity. We hypothesize that the presence of immunosuppressive plasma exosomes in refractory/relapsed AML patients may impair anti-tumor activity of adoptive cell therapies.

## Methods

Venous blood (20-50 mL) was obtained from patients with refractory/relapsed AML (n=7). Exosome fractions were isolated from the patients' plasma and plasma of normal controls by using mini-size exclusion chromatography with Sepharose 2A. Protein levels, numbers and the size of exosomes (qNano) and their morphology (transmission electron microscopy) were determined. Exosomes were characterized by Western blots for expression of exosome markers, Tsg101 and CD81, and myeloid cell-surface markers associated with AML, interleukin-3 receptor alpha chain (CD123) and C-type lectin-like molecule-1 (CLL-1), CD44, CD96 and TGF-β1. Isolated normal human NK cells were co-incubated with AML exosomes and multiparameter flow cytometry was used to monitor changes in expression levels (mean fluorescence intensity) of NKG2D on NK cells.

## Results

Exosome fractions isolated from AML patients' plasma with refractory/relapsed AML had mean protein content of 36 µg protein/mL plasma. AML exosomes contained blast markers, CD123, CLL-1, CD44, CD96 suggesting their leukemia origin. They were also enriched in TGF-β1, which is known to interfere with NK cell activity and promote Treg expansion. Co-incubation of AML exosomes with activated NK cells resulted in down-regulation of NKG2D expression with a concomitant reduction of NK-cell cytotoxicity. Antibody neutralization of TGF-β1 carried by AML exosomes significantly abrogated their immunosuppressive activity.

## Conclusions

The persistently elevated levels of biologically-active TGF-β1+ exosomes carrying leukemia blast markers in plasma of refractory/relapsed AML patients indicate that existing immune suppression will interfere with anti-leukemia effects of adoptive cell therapies.

